# Locust Bean Gum: A Natural Polysaccharide as an Eco-Friendly Corrosion Inhibitor for N80 Carbon Steel in CO_2_-Saturated Saline Solution, Useful for the Oil and Gas Industry

**DOI:** 10.3390/molecules30234534

**Published:** 2025-11-24

**Authors:** Gaetano Palumbo, Marcin Górny, Dominika Święch, Adarsh Rai, Mahmoud M. Youssif

**Affiliations:** 1 Faculty of Foundry Engineering, AGH University of Krakow, al. A. Mickiewicza 30, 30-059 Krakow, Poland; mgorny@agh.edu.pl (M.G.); dswiech@agh.edu.pl (D.Ś.); 2 Faculty of Metals Engineering and Industrial Computer Science, AGH University of Krakow, al. A. Mickiewicza 30, 30-059 Krakow, Poland; rai@agh.edu.pl; 3 Faculty of Non-Ferrous Metals, AGH University of Krakow, al. A. Mickiewicza 30, 30-059 Krakow, Poland; youssif@agh.edu.pl; 4 Department of Chemistry, Faculty of Science, Tanta University, Tanta 31527, Egypt

**Keywords:** locust bean gum, sweet corrosion, corrosion inhibition, carbon steel N80

## Abstract

This study evaluated locust bean gum (LBG), a polysaccharide thickening agent, as an anti-corrosion active compound against sweet corrosion for N80 carbon steel used in the oil and gas sector. The assessment involved weight loss and electrochemical measurements at different temperatures (e.g., 25 °C and 80 °C) and immersion durations (up to 168 h) in a CO_2_-saturated 2 wt.% KCl solution. The electrochemical results showed that LBG effectively inhibited sweet corrosion at both temperatures, and its efficacy increased with its concentration, reaching maximum inhibition efficiency of 84.11% at 25 °C and 55.81% at 80 °C, using 0.3 g L^−1^ of LBG after 24 h of immersion. At 25 °C, and with 0.3 g L^−1^ of LBG, the inhibition action of LBG did not change, even after 168 h of immersion (e.g., 83.97%). At 80 °C, LBG showed a good inhibition up to 72 h (e.g., 47.04%), after which LBG had no additional protective effect. This result is attributed to the formation of a FeCO_3_ layer that covered the entire metal surface, blocking the adsorption of LBG. Potentiodynamic tests revealed that LBG’s inhibitory effect is of a mixed type. The Temkin adsorption isotherm model accurately described the data, indicating that LBG adsorption involves primarily physical interactions, with some chemical contributions. Activation energy and heat of adsorption calculations support the physical nature of LBG’s adhesion. FTIR analysis confirmed the interaction between LBG and N80 carbon steel, while SEM-EDS provided visual evidence of LBG’s influence on the metal surface.

## 1. Introduction

Oil extraction typically occurs in three recovery stages [1]. Literature reports that during the first two stages (e.g., primary and secondary recovery), approximately 20 to 40% of the original-oil-in-place (OOIP) is recovered [1]. After these initial stages, a third stage (e.g., tertiary recovery) is used to recover the remaining OOIP. This stage is also known as Enhanced Oil Recovery (EOR). CO_2_ flooding is one of the most commonly used EOR methods. Among the various CO_2_-EOR methods, water-alternating CO_2_ injection is the most effective method. It involves alternating water and CO_2_ injections into a depleted reservoir to boost oil production [1].

N80 carbon steel, as well as similar alloys such as K55 and L80, is widely used in the petroleum sector for its mechanical properties and affordability [2,3]. However, dissolved carbon dioxide forms weak carbonic acid, which can weaken steel infrastructures [3,4,5,6]. Over the last decade, various methods have been employed to protect metal surfaces and minimize the risk of sweet corrosion. Adding corrosion inhibitors (CIs) remains one of the most cost-effective options [3,4,5,6]. CIs are substances that, once dissolved in the corrosive solution, adsorb onto the metal surface and form a protective layer. CIs such as long-chain imidazole and imidazolines, and their condensation products, are frequently used in the petroleum industry [5,7,8,9,10,11,12,13]. They are heterocyclic substances containing heteroatoms (e.g., N, O, or S) and are reported to be excellent CIs due to their multiple absorption sites [5,7,8,9,10,14]. However, all of these compounds are environmentally toxic, harmful, and expensive [7,10]. Additionally, these nitrogen-containing compounds are known to be somewhat unstable during storage and often emulsify in formation water [10]. The petroleum industry now emphasizes strict adherence to the Paris Commission (PARCOM) regulations concerning the use of sweet CIs. The guidelines state that an inhibitor must not bioaccumulate, be non-toxic, and be readily biodegradable [2,8,10]. Therefore, due to these limitations, scientists have focused on greener and less expensive alternative CIs.

Naturally occurring compounds (e.g., plant-based extracts and bio-polymers) have gained significant popularity as affordable CIs against corrosion [2,3,4,15,16,17,18,19,20,21,22,23,24,25,26,27,28,29]. Among these natural substances, polysaccharides have been recognized as effective CIs [2,3,4,15,16,17,18,19,21,22,27,28]. Polysaccharides, such as chitosan, guar gum, xanthan gum, and others, are rich in functional groups containing heteroatoms, which can act as adsorption centers. Furthermore, because they consist of long molecular chains, they can protect a larger surface area through their multiple adsorption centers, at the same concentration, compared to their monomeric counterparts [15,18,28]. Therefore, polysaccharides are expected to be more effective at preventing corrosion than small plant-based extract molecules. However, these studies have focused on using these natural compounds as a CI in strong acid media (e.g., HCl, H_2_SO_4_) [15,16,17,18,19]. In contrast, a few studies have examined the use of naturally occurring (e.g., unmodified) CIs to prevent sweet corrosion [2,3,4,27]. Recent years have seen an increasing number of studies of modified carbohydrate compounds as CIs to mitigate CO_2_ corrosion [30,31,32,33]. Chemical grafting, esterification, and etherification are all methods used to improve the anti-corrosion properties of these naturally occurring carbohydrates [33]. However, these methods require solvents, operate at high temperatures, and are time-consuming. Furthermore, the addition of strong adsorbent groups may impart toxic effects on the natural compounds.

This research group, committed to developing sustainable CIs, investigates, for the first time, the use of locust bean gum (LBG) as a green alternative CI for protecting N80 carbon steel against sweet corrosion. LBG is a high molecular weight natural polysaccharide refined from carob seeds (Ceratonia siliqua tree, primarily located in the Mediterranean regions) [34,35]. Given its biodegradability, biocompatibility, and non-toxicity, LBG is frequently used in the food industry as a thickening and stabilizing agent [34,35]. Moreover, LBG shows a strong resistance to changes in temperature, pH, and salinity [35]. Due to these characteristics, LBG has found a new role as a thickening agent in recently developed, greener EOR methods that use more environmentally friendly drilling solutions [1]. Recently, new studies have highlighted that LBG can also be a good, eco-friendly CI for steel infrastructure [16,36,37]. Jano et al. [37] investigated the effect of LBG in a chloride-containing 1 M H_2_SO_4_ solution at 25 °C and observed an inhibition efficiency of around 81%. Abbout et al. [16] studied the effect of this natural compound in a 1 M HCl solution, reporting an inhibition efficiency of 87% at 25 °C after 30 min of immersion with 1.0 g L^−1^ of LBG. As shown, these studies have focused on using LBG as a CI in strong acid media for the metal pickling process, whereas no studies have assessed its use as a CI to inhibit CO_2_ corrosion. Sweet corrosion is considered more harmful than that caused by strong acids due to its buffering effect, caused by carbonic acid produced when CO_2_ dissolves in water [38]. The duration of the metal pickling process can range from 30 to 90 min; therefore, these studies have tested the inhibition action of LBG for only a limited time, up to 1 h, and at low temperatures (e.g., 40 °C). On the other hand, the EOR injection process can take several days, and the reservoir temperature is much higher [39]. At high temperatures (e.g., above 60 °C [40]), in the presence of CO_2_, FeCO_3_ forms on the metal surface, which can significantly affect both the metal’s corrosion resistance and the inhibitor’s adsorption. Many studies on the use of natural CIs were frequently conducted at temperatures below the FeCO_3_ formation threshold and/or with short immersion times. In these cases, understanding the practical impact of the inhibitor may be difficult. Sometimes, pipelines are often in use for several years before CIs are added. As a result, their internal surfaces could already be covered with various types of scale, such as uncorroded iron carbide and/or iron carbonate [41]. Thus, to properly assess the CI’s performance in the presence of CO_2_, it is crucial to consider its behavior when FeCO_3_ partially or fully covers the steel surface. Therefore, this study aimed to prove that the thickening agent LBG used in the EOR process can also serve as a practical, eco-friendly active component against CO_2_ corrosion. The investigation was carried out in a CO_2_-saturated 2% KCl solution by weight-loss measurements, electrochemical methods (e.g., EIS and PDP), and surface morphological analysis (e.g., SEM-EDS, XRD, and FTIR). The present study was designed to understand the interaction of the inhibitor with bare and partially or fully corrosion-scale-formed layers on the N80 carbon steel surface in a CO_2_-saturated 2% KCl solution.

## 2. Results

### 2.1. Weight Loss Measurements

**Figure 1:**
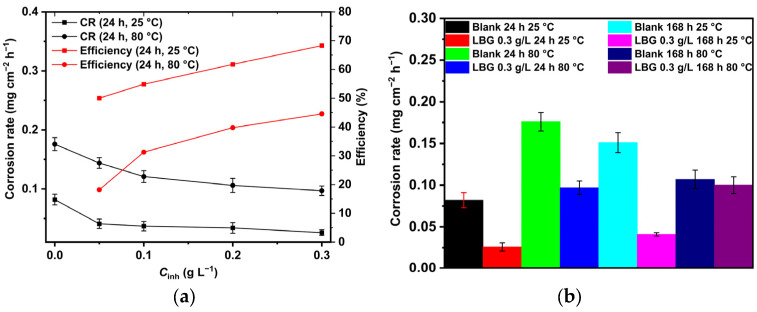
(**a**) Average weight loss and inhibition efficiency after 24 h without and with different concentrations of LBG. (**b**) Average weight loss after 168 h without and with 0.3 g L^−1^ of LBG.

Figure 1a and Table S1 (Supplementary Materials) show the average weight loss (AWL) and inhibition efficiency (IE%) observed after 24 h of immersion with varying concentrations of LBG at 25 °C and 80 °C. The data clearly indicate that LBG reduces steel corrosion, and its efficacy increases with its concentration, reaching a maximum IE of 68.29%. At 80 °C, although the AWL increases, LBG still offers a notable level of protection against sweet corrosion, reaching a maximum IE of 44.59% at a concentration of 0.3 g L^−1^. Figure 1b shows the AWL as a function of time. At room temperature, the corrosion product formed on the metal surface is porous and not protective [40,42]; therefore, as the exposure time increases, the AWL increases as well, going from 0.082 to 0.151 mg cm^−2^ h^−1^, respectively, after 24 and 168 h of exposure (Table S2). By contrast, in the presence of LBG, even if AWL increases, it is more contained (e.g., from 0.026 to 0.041 mg cm^−2^ h^−1,^ after 24 and 168 h, respectively), with an IE reaching a maximum of 72.85 at an LBG concentration of 0.3 g L^−1^. However, as immersion time increases, the formation of a protective FeCO_3_ layer at 80 °C becomes more favorable [40,42]. It follows from the data that AWL decreased after 168 h, and the effectiveness of LBG vanishes because of this protective layer (Table S2).

Table S3 proves that, compared to other natural corrosion inhibitors, LBG is a reliable and eco-friendly choice for preventing long-term sweet corrosion of carbon steel.

### 2.2. Adsorption Study and Activation Parameters

**Figure 2:**
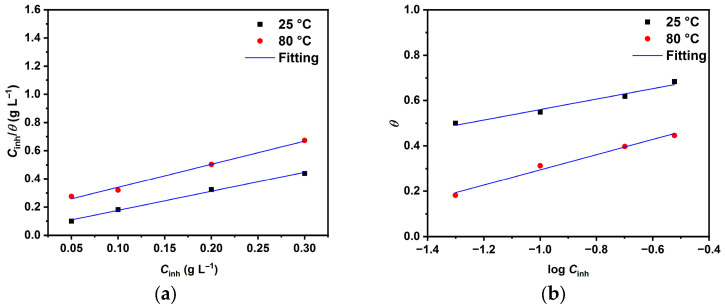
Adsorption models (**a**) Langmuir, and (**b**) Temkin.

To better understand the adsorption process, several adsorption isotherms were used to match the weight-loss results. The Langmuir (Equation (1)) [3,15,16,18] and Temkin (Equation (2)) models [16,17,20], have shown the best fit, as illustrated in Figure 2.

(1) $$\frac{C_{\mathrm{inh}}}{\theta }=\frac{1}{K_{\mathrm{ads}}}+C_{\mathrm{inh}}$$

(2) $$\exp(-2a\theta )=K_{\mathrm{ads}}C_{\mathrm{inh}}$$

Here, *K*_ads_ represents the constant related to adsorption energy, *C*_inh_ is the inhibitor concentration, *a* is the adsorbate interaction factor, and *θ* is the surface coverage [15,18]. Adsorption isotherms can be used to determine *K*_ads_, which is in turn related to the free energy of adsorption (${∆G}_{\mathrm{ads}}^{{}^{\circ}}$) [3,19]:

(3) $$∆G_{\mathrm{ads}}^{{}^{\circ}}=-\mathrm{RT}\ln(10^{3}\;K_{\mathrm{ads}})$$

where the value 1 × 10^3^ represents the concentration (g L^−1^) of water molecules in solution.

Table 1 presents the adsorption isotherm parameters, showing that the Langmuir isotherm produces higher *R*^2^ values across the tested temperatures. This suggests it is the most appropriate model for describing LBG adsorption on the metal surface. Similar findings have been reported in the literature where polysaccharides served as a CI in different solutions [3,4,16,17,18,19]. Nevertheless, selecting an isotherm should rely not only on *R*^2^ values but also on the model’s assumptions. For instance, the Langmuir model assumes (I) no interaction between adsorbed molecules and (II) a fixed number of adsorption sites on the metal surface, each holding one adsorbed molecule [43,44]. Since *R*^2^ deviates from 1, this likely indicates interactions among adsorbed LBG molecules on the surface [21]. Consequently, the Temkin isotherm, which incorporates the interaction parameter ignored by Langmuir, was used to fit the WL measurements [4,21,44]. Table 1 shows that all values of ${∆G}_{\mathrm{ads}}^{{}^{\circ}}$ are negative, indicating that the adsorption of LBG is a spontaneous process. The adsorption process can be considered a combination of chemical and physical adsorption. Furthermore, the negative values of *a* at both temperatures suggest repulsive forces among the adsorbed macromolecules [4,16,17]. These results align with previous studies [3,15,16,18,19,36].

The simplified Arrhenius Equation can describe the dependence of the corrosion rate on temperature (Equation (4)) [4,5]:

(4) $$\log\frac{{\mathrm{CR}}_{2}}{{\mathrm{CR}}_{1}}=\frac{E_{a}}{2.303R}\left(\frac{1}{T_{1}}-\frac{1}{T_{2}}\right)$$

where CR_1_ and CR_2_ are the corrosion rates at *T*_1_ (25 °C) and *T*_2_ (80 °C), respectively. *E*_a_ is the apparent activation energy. The heat of adsorption (*Q*_ads_) parameters were determined using Equation (5) [4,5]:

(5) $$Q_{\mathrm{ads}}=2.303R\left[\log\left(\frac{{\;\theta }_{2}}{1\;-{\;\theta }_{2}}\right)-\log\left(\frac{\theta_{1}}{1\;-\theta_{1}}\right)\right]\left(\frac{T_{1}\;\times \;T_{2}\;}{T_{2}\;-\;T_{1}}\right)$$

Here, *θ*_1_ and *θ*_2_ are the degrees of surface coverage at *T*_1_ (25 °C) and *T*_2_ (80 °C). The computed parameters are shown in Table 2.

The literature reports that if the *E*_a_ values in the presence of the inhibitor are higher than those observed for the blank sample, the adsorption process is considered a physical adsorption. In contrast, lower or equal *E*_a_ values indicate that the adsorption is a chemical [3,4,19]. Table 2 shows that, for the inhibited sample, the values of *E*_a_ are higher than those for the uninhibited sample. This may be because of the LBG adsorptive layer, which decreases the surface area exposed to corrosion [3,4,19]. Also, *E*_a_ increases with LBG concentration, suggesting that the activation energy mainly governs the process [18,19]. These observations, together with the ${∆G}_{\mathrm{ads}}^{{}^{\circ}}$ values, strengths, the proposed mixed-type adsorption process (i.e., physical and chemical adsorption) [16,36].

### 2.3. Electrochemical Measurements

**Figure 3:**
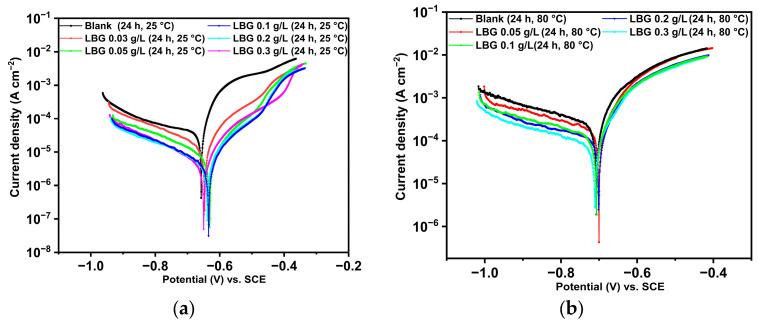
Potentiodynamic polarization plots with and without concentrations of LBG after 24 h of immersion time at: (**a**) 25 °C and (**b**) 80 °C.

**Figure 4:**
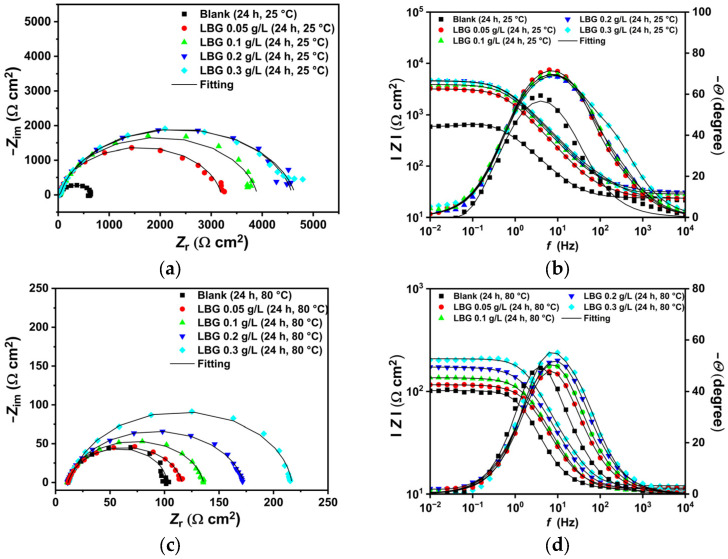
EIS plots in the absence and presence of different concentrations of LBG after 24 h of immersion time at: (**a**,**b**) 25 °C and (**c**,**d**) 80 °C.

The potentiodynamic polarization experiments performed after exposing the sample to the tested condition for 24 h, both with and without LBG, are presented in Figure 3, with the kinetic parameters listed in Table 3. The results indicate that at 25 °C (Figure 4a), the addition of LBG affects the corrosion current density (*i*_corr_), which decreases as the LBG concentration increases, achieving a maximum corrosion IE of 87.35% at 0.3 g L^−1^ of LBG. Notably, the anodic branches lack a clear Tafel region; thus, *i*_corr_ values were estimated by extrapolating the cathodic Tafel region, consistent with Amin et al. [45].

At 80 °C, the PDP plots show similar features to those at 25 °C, with *i*_corr_ values decreasing as the LBG concentration rises (IE = 47.32%). Particularly, LBG significantly affects the cathodic reaction, suggesting it is more prone to adsorbing on cathode sites and inhibiting the hydrogen evolution reaction (HER), as elaborated in more detail in Section 3.3. IE% was observed to decrease as temperature increased across all concentrations, which supports the proposed adsorption mechanism involving physisorption. At both temperatures, the corrosion potential (*E*_corr_) values in the presence of LBG shifted toward more negative values compared to the solution without the inhibitor, although this shift was minimal (e.g., less than 0.085 V [3]). This result shows that LBG acts as a mixed-type inhibitor, consistent with previous findings reported for gum-like corrosion inhibitors [3,4,17,18]. The PDP results are consistent with those observed from the gravimetric measurements.

The EIS plots obtained at 25 °C and 80 °C are presented in Figure 4. The Nyquist spectrum for the uninhibited sample at 25 °C (Figure 4a) features a wide, depressed arc from high (HF) to medium (MD) frequencies, with an inductive loop at low frequency (LF). With the addition of LBG, the depressed semicircle widens and the inductive loop vanishes, likely shifting to lower frequencies outside the studied range. This disappearance is followed by the emergence of a second time constant in the HF region (between 10^4^ and 10^2^ Hz, Figure 4b), in addition to the charge-transfer time constant and the double-layer capacitance. Various researchers have reported similar findings, which can be attributed to the adsorption of the inhibitor, forming an adsorptive layer that slows down both the cathodic and anodic reactions [3,10,11].

At 80 °C, the Nyquist plots show that, regardless of the presence of LBG, all impedance spectra display a broad, depressed arc from high (HF) to medium (MD) frequencies. The EIS spectra show a pattern similar to that observed at 25 °C, with the capacitive loop linked to the charge transfer resistance gradually increasing as the LBG concentration rises.

**Figure 5:**
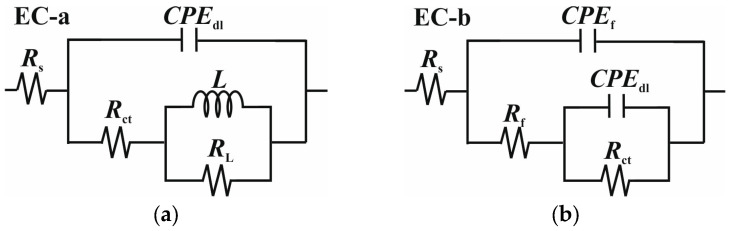
Equivalent circuits used to fit experimental data. (**a**) without LBG at 25 °C; (**b**) with LBG at 25 °C, and without and with LBG at 80 °C.

The data were modeled using the equivalent circuits (EC) depicted in Figure 5, based on findings from similar studies in the literature [2,3,10,11,27]. The explanation of the EC is reported in the Supplementary Materials. The fitting parameters and the chi-squared (*χ*^2^) values are listed in Table 4. In the EC proposed, the *CPE* elements replaced capacitance elements (*C*) to depict the inhomogeneity of the corroded surface. The impedance of a *CPE* includes two parameters: *Y*_o_ and *n* (Equation (S1)). *Y*_o_ represents the capacitance, while *n* shows how much the behavior deviates from an ideal capacitor, also indicating the surface’s smoothness [10]. Based on the model proposed by Brug et al. [46], *CPE*_dl_ can be used to calculate the double-layer capacitance (*C*_dl_) (Equation (S2)) [47].

### 2.4. Effect of Time

**Figure 6:**
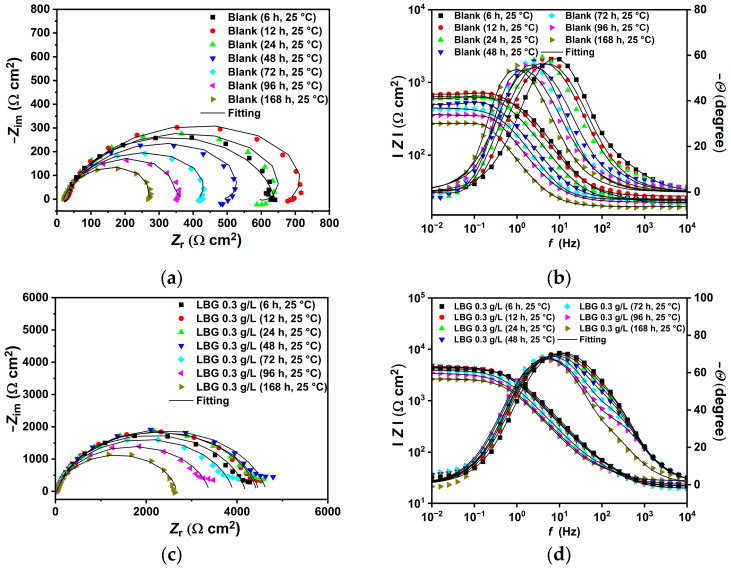
EIS plots carried out without (**a**,**b**) and with the presence of 0.3 g L^−1^ of LBG (**c**,**d**) at 25 °C at different immersion times.

**Figure 7:**
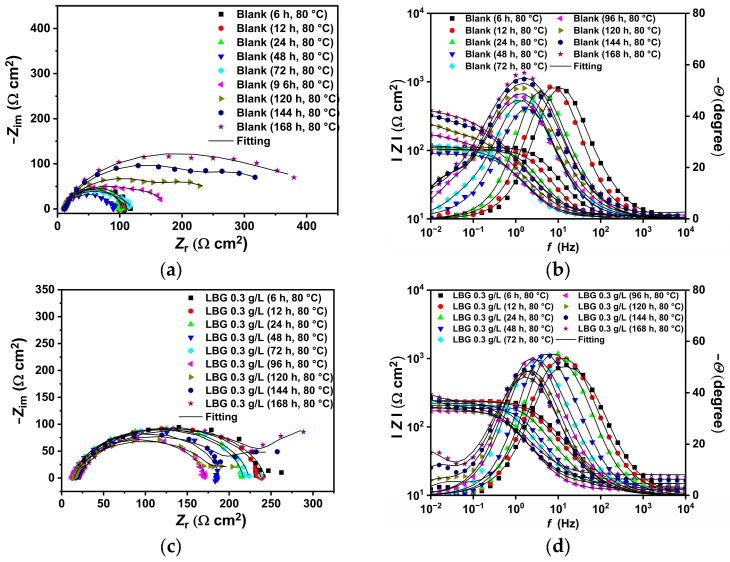
EIS plots carried out without (**a**,**b**) and with the presence of 0.3 g L^−1^ of LBG (**c**,**d**) at 80 °C at different immersion times.

Figure 6 and Figure 7 display the EIS measurements taken at various immersion times (up to 168 h), both with and without 0.3 g L^−1^ of LBG, at 25 and 80 °C. The plots were fitted with ECs shown in Figure 5, and the resulting parameters are presented in Tables S4 and S5. According to the data, at 25 °C, LBG shows its effectiveness within the first 6 h of the test, with an IE of around 85%. After which, IE remains constant throughout the experiment. This indicates that LBG rapidly adheres to the metal surface, providing fast, long-lasting protection.

At 80 °C, the inhibitory effect of LBG decreases, possibly because it desorbs from the metal surface. This result confirms that LBG undergoes a physical adsorption process on the steel surface. That being said, LBG can still help reduce sweet corrosion to some degree, even at higher temperatures. IE% remains stable for up to 72 h, then drops to nearly 0% with extended exposure.

### 2.5. Surface Analysis

**Figure 8:**
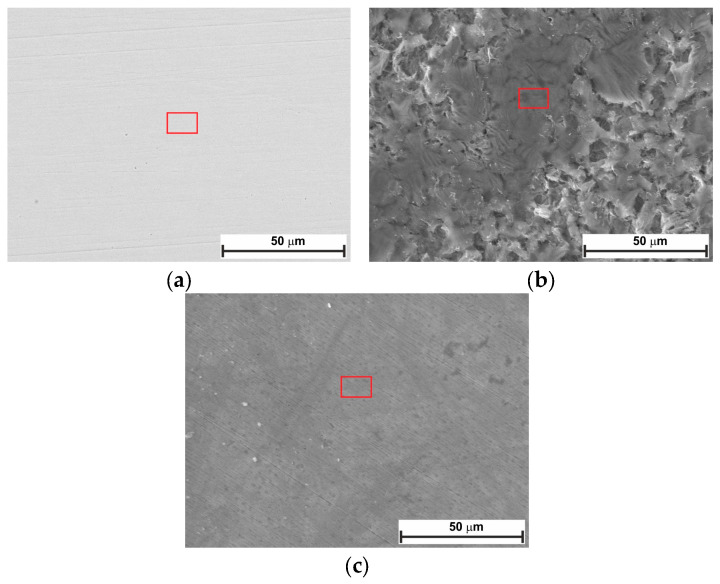
SEM and EDS (red square) analysis of the tested steel surface after 24 h of immersion at 25 °C: (**a**) polished sample; (**b**) without, and (**c**) with 0.3 g L^−1^ of LBG solution.

**Figure 9:**
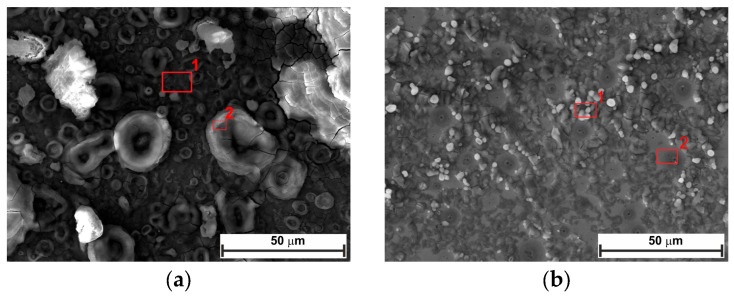
SEM and EDS (red square) analysis of the tested steel surface after 168 h of immersion at 25 °C: (**a**) without, and (**b**) with 0.3 g L^−1^ of LBG solution.

The surface of the N80 carbon steel sample was characterized after 24 and 168 h at OCP, both with and without 0.3 g L^−1^ of LBG. Surface analysis was conducted using SEM-EDS and XRD. Figure 8 compares the top-view SEM images for the polished, blank, and inhibited samples exposed to the tested solution after 24 h of immersion at 25 °C. The top-view image of the uninhibited sample shows a sharp contrast compared to the inhibited sample. Based on the pictures, corrosion products appeared on the surface in the absence of LBG, whereas no signs of corrosion were observed, and polishing scratches are still visible after 24 h of immersion. The EDS analysis of the samples presented in Figure S1 shows a lower percentage of Fe and a higher percentage of O elements in the absence of LBG. The greater loss in Fe wt.% can be attributed to the increased corrosion activity of N80 steel, resulting in the subsequent formation of oxide-containing corrosion products when LBG is not present [8]. As exposure time increases, corrosion rates increase. From Figure 9, it can be seen that the sample without LBG is entirely covered by a corrosion layer containing a high percentage of O elements (EDS analysis, Figure S2a,b). In contrast, the sample exposed to the solution containing the inhibitor is only partially covered by a corrosive layer, with large areas of still-intact metal (EDS analysis, Figure S2c,d).

**Figure 10:**
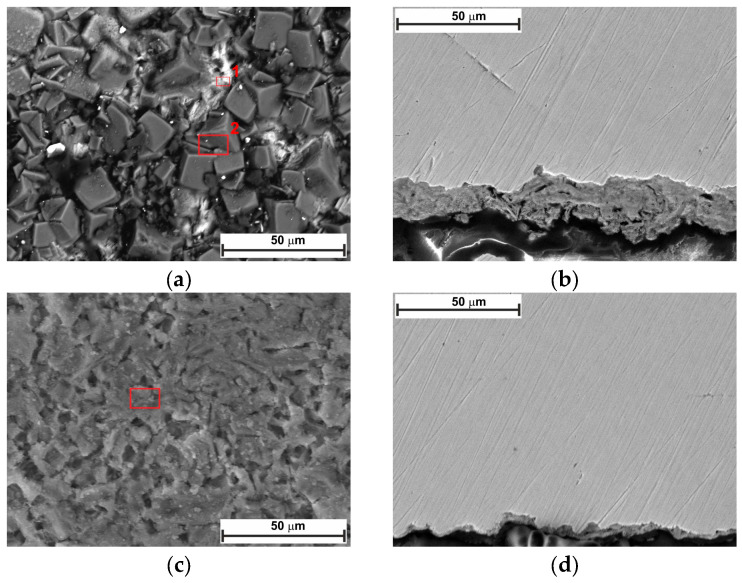
SEM and EDS (red square) analysis of the tested steel surface after 24 h of immersion at 80 °C: (**a**,**b**) without, and (**c**,**d**) with 0.3 g L^−1^ of LBG solution.

**Figure 11:**
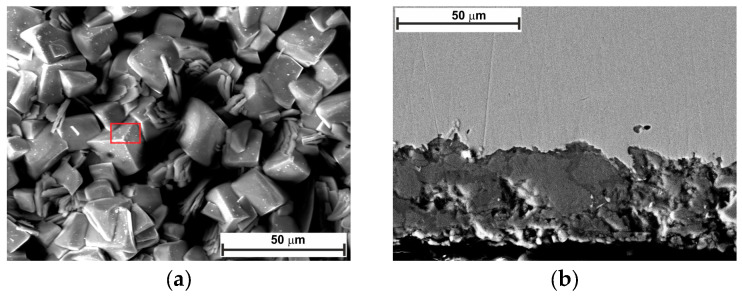

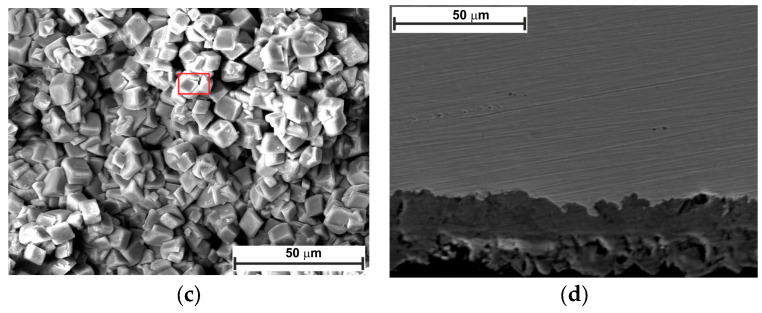
SEM and EDS (red square) analysis of the tested steel surface after 168 h of immersion at 80 °C: (**a**,**b**) without, and (**c**,**d**) with 0.3 g L^−1^ of LBG solution.

**Figure 12:**
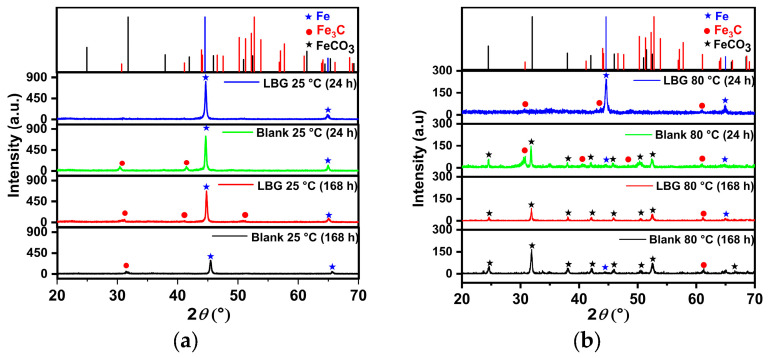
GIXRD analysis performed at different immersion times: (**a**) at 25 °C, and (**b**) at 80 °C. (FeCO_3_, ICDD:29-0696; Fe_3_C, ICDD:35-0772; Fe, ICDD:06-0696).

Figure 10 and Figure 11 show the top-view and cross-section SEM analysis of the sample surfaces with or without LBG 0.3 g L^−1^ at 80 °C, after 24 and 168 h of immersion, respectively. After 24 h of immersion, the surface of the uninhibited sample shows FeCO_3_ crystals on a porous Fe_3_C layer (EDS analysis, Figure S3). No sign of FeCO_3_ crystals is visible on the surface of the inhibited sample. This phenomenon may be due to LBG’s inhibitory action, which reduces metal corrosion and thereby delays the formation of FeCO_3_ crystals. Cross-sectional images confirm LBG’s inhibitory effect. Increasing the immersion time results in both the uninhibited and inhibited samples being covered by a layer of FeCO_3_. The XRD results agree with the SEM analyses and indicate that at room temperature (Figure 12a), no FeCO_3_ crystal peaks are observed. At 80 °C, all remaining samples show peaks related to FeCO_3_ crystals, except for the inhibited sample exposed after 24 h.

## 3. Discussion

### 3.1. Low Temperature (T = 25 °C)

**Figure 13:**
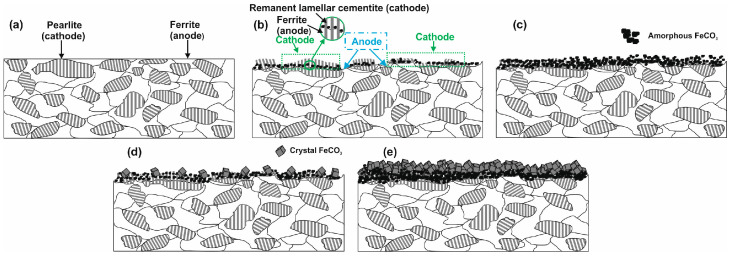
Schematic diagrams illustrating the corrosion process on carbon steel N80 in CO_2_-saturated saline solution. (**a**) Polished metal; (**b**,**c**) 24 and 168 h of immersion at T = 25 °C; (**d**) 24 h of immersion at T = 80 °C for the blank solution; (**e**) 168 h of immersion at T = 80 °C.

Figure 13 displays a schematic diagram illustrating the corrosion mechanism of N80 carbon steel in CO_2_-containing environments, which can help understand the corrosion phenomenon at different temperatures. Alloy N80 is a ferrite-perlite steel, with the ferrite component accounting for around 41% of the total microstructure. The perlite phase has a lamellar structure with cementite (Fe_3_C) embedded within a ferrite matrix (Figure S5). Gravimetric experiments revealed an AWL of 0.082 mg cm^−2^ h^−1^ after 24 h of immersion (Figure 1a). Corrosion occurred as a result of the galvanic corrosion between the nobler cementite structure and the surrounding ferrite matrix (Figure 13a). This process caused the ferrite phase to dissolve, with the Fe_3_C exposed on the surface (Figure 13b). The results show that LBG improves the corrosion resistance of N80 steel, and that its effectiveness increases by increasing its concentration. At the optimal LBG concentration (e.g., 0.3 g L^−1^), the AWL was 0.026 mg cm^−2^ h^−1^, which corresponds to an IE of 68.20%. SEM analysis shown in Figure 8c confirmed the goodness of LBG against sweet corrosion. Unlike the uninhibited solution (Figure 8b), in the presence of 0.3 g L^−1^ LBG, the metal surface appears noticeably smoother. However, abrasive scratches remain visible, indicating that the adsorption of LBG reduced the corrosion of the metal. The EDS analysis further validated the protective role of the LBG. According to the EDS analysis shown in Figure S1b,c, for the uninhibited and inhibited solutions, a lower percentage of Fe and a higher percentage of O elements are observed in the absence of LBG. The greater loss in Fe wt.% can be attributed to the increased corrosion activity of N80 steel, resulting in the subsequent formation of oxide-containing corrosion products when LBG is not present (Figure 13c) [8].

In an aqueous solution, CO_2_ gas forms carbonic acid [48]:

(6) $$CO_{2}+H_{2}O_{\left(l\right)}↔H_{2}CO_{3}$$

This, in turn, dissociates into bicarbonate and then carbonate anions:

(7) $$H_{2}CO_{3}↔{{\mathrm{HCO}}_{3}^{-}+H}^{+}$$

(8) $${\mathrm{HCO}}_{3}^{-}↔CO_{3}^{2-}+H^{+}$$

The potentiodynamic polarization experiments (Figure 3a and Table 3) have shown that *i*_corr_ values decreased, and that both cathodic and anodic reactions are inhibited in the presence of LBG. However, the profiles of the anodic and cathodic curves change in the presence of LBG, indicating that the inhibitor alters the corrosion mechanism process. For instance, the cathodic region of the uninhibited sample shows a diffusion-controlled reaction. This process is common in CO_2_-saturated environments and results from the non-uniform reaction of CO_2_ hydration on the steel surface (Equation (6)) [5,49,50]. In contrast, the anodic branch shows active dissolution. On the other hand, adding LBG seems to place both curves more under activation control.

In a CO_2_-saturated solution, the corrosion process is controlled by the following cathodic reactions, which are, in turn, pH-dependent [48]:

(9) $${2H}^{+}+2e^{-}\to H_{2}\;\;\;\;\;\;\;\;\;\;\;\;\;\;\;\;\;\;\;\;\left(pH<4\right)$$

(10) $$2H_{2}CO_{3}+2e^{-}\to {2{\mathrm{HCO}}_{3}^{-}+H}_{2}\;\;\;\;\;\;\;\;(4<pH<6)$$

(11) $$2{\mathrm{HCO}}_{3}^{-}+2e^{-}\to 2CO_{3}^{2-}+H_{2}\;\;\;\;\;\;\;\;\;\;\;\;\;\;\;\;\;\;\;\;\;\;(4<pH<6)$$

(12) $$2H_{2}O+2e^{-}\to 2OH^{-}+H_{2}\;\;\;\;\;\;\;\;\;\;\;\;\;\;\;\;\;\;\;\;\;\;\;\;\;\;\;\;(pH>9)$$

The dissolution of iron occurs according to the following reactions [51]:

(13) $$\mathrm{Fe}+H_{2}O\to {\mathrm{FeOH}}_{\left(\mathrm{ads}\right)}+{H^{+}+e}^{-}$$

(14) $${\mathrm{FeOH}}_{\left(\mathrm{ads}\right)}\to {\mathrm{FeOH}}^{+}+e^{-}$$

(15) $${\mathrm{FeOH}}^{+}+H^{+}\to {\mathrm{Fe}}^{2+}+H_{2}O$$

The pH of the solution was 3.5 at the beginning of the experiments, which rose to 5.50 after 24 h of immersion; therefore, the findings indicate that the presence of LBG inhibits both cathodic (Equations (9)–(11)) and anodic dissolution (Equations (13)–(15)) reactions, as evidenced by the decrease observed in both the cathodic and anodic branches.

The EIS experiments displayed in Figure 4 also confirm that the inhibitor influences the corrosion mechanism. The blank sample shows an inductive loop at LF, which is known to be linked with the adsorption of intermediate species such as FeOH_(ads)_ [9,49,52,53,54]. As shown in Figure 4a, in the presence of LBG, the indicative loop disappears; however, a second time constant in the HF region (between 10^4^ and 10^2^ Hz) appears (Bode plot, Figure 4b). In the consecutive mechanism proposed by Bockris, water binds to the bare iron surface, enabling the formation of an intermediate FeOH_(ads)_ as the initial step in iron dissolution. After adsorption, the steel corrodes, releasing Fe^2+^ ions into solution, which in turn, react with other ions in the electrolyte to form corrosion products that precipitate on the metal surface. In the presence of LBG, no inductive loop was observed. This phenomenon may be ascribed to the inhibitor adsorbing onto the metal surface, removing water molecules from the surface, and thereby hindering the formation of FeOH_(ads)_. The effectiveness of the inhibitor is also evident from the increase in *R*_ct_ (e.g., charge transfer resistance) with its values ranging from 725.05 Ω cm^2^ for the blank sample to 4565.50 Ω cm^2^ for the inhibitor sample, achieving a maximum IE of 84.11% at a LBG concentration of 0.3 g L^−1^. Higher *R*_f_ (e.g., film resistance) values also demonstrate the inhibitory property of LBG. In contrast, *C*_dl_, which is inversely proportional to *R*_ct_, decreases from 215.59 to 9.17 µF cm^−2^ for the blank and the inhibited samples, respectively, indicating a reduction in the active surface area where the cathodic reaction takes place [53]. Based on the Helmholtz model, this decrease is attributed to a change at the solution/metal interface resulting from the replacement of water molecules with LBG molecules. The parameter *n*_dl_ is another valuable metric to evaluate the inhibitor’s effectiveness. The parameter is linked to the metal surface’s roughness; a low value indicates a rough surface, while a higher value indicates a smoother surface. According to the data, after adding LBG, the *n*_dl_ values increase gradually with increasing inhibitor concentration. This suggests a smoother surface due to the inhibitor’s adsorption at the steel surface’s adsorption sites, as confirmed by SEM analysis shown in Figure 8a,b, which compare the blank and inhibited samples, respectively.

The effectiveness of LBG against sweet corrosion was also examined over an extended immersion period. Figure 6 shows the EIS experiments conducted after different immersion times, both with and without the optimal LBG concentration (e.g., 0.3 g L^−1^). The data show that the *R*_ct_ of the sample immersed in the blank solution steadily decreases over time, from 600 Ω cm^2^ after 6 h to 257 Ω cm^2^ after 168 h. Over time, due to the preferential dissolution of the ferrite matrix, cementite lamellas build up on the surface. As mentioned before, the cementite potential is higher (e.g., nobler) than that of the surrounding ferrite matrix; therefore, the continuous growth of the cementite area leads to an increase in the cathodic to anodic area ratio. The increase in cathodic activity is evident from the rise in *C*_dl_ from 72 to 1533 µF cm^−2^. *C*_dl_ relates to the surface area available for cathodic reactions; an increase in Fe_3_C surface area increases its magnitude. Thus, as the surface area of Fe_3_C grows over time, the cathodic reactions (Equations (9)–(11)) govern the interfacial process, and the metal’s dissolution accelerates (Figure 13b) [4,55,56]. The increase in cathodic activity is also evident from the SEM analysis performed after 168 h of immersion, shown in Figure 9a. The image shows that the corrosion layer covering the entire metal surface clearly exhibits signs of ruptures. The ruptures are probably due to HER at the cathodic sites. The accumulation of this gas leads to physical disruption of the thin corrosion layer, thus further exposing the metal surface to the aggressive solution (EDS analysis, Figure S2a,b). By contrast, as shown in Figure 6c,d, although the metal still corrodes in the presence of LBG over time, its dissolution rate is significantly reduced by LBG absorption. After 168 h of immersion at 0.3 g L^−1^, the IE was calculated to be 83.97%. The SEM analysis shown in Figure 9b confirms the high inhibition efficiency observed after 168 h of immersion. The image indicates that the inhibited sample is only partially coated with a thin layer of corrosion products (EDS analysis, red square 1, Figure S2c). At the same time, large portions of the metal remain uncorroded (EDS analysis, red square 2, Figure S2d). The findings suggest that LBG inhibits ferrite dissolution and increases metal protection over prolonged immersion. The findings are confirmed by the GIXRD results reported in Figure 12. The GIXRD spectra show only diffraction peaks from the iron and cementite phases, indicating that the corrosion product formed on the steel was mainly an amorphous compound (Figure 13c). As expected, no traces of FeCO_3_ crystals were observed, even after prolonged immersion times (e.g., 168 h). The GIXRD findings agree with those reported in the literature, indicating that no FeCO_3_ crystals form at temperatures below 60 °C [40,42]. Previous studies reported a similar result [57,58]. The authors observed that at this temperature and pH, only an amorphous-like FeCO_3_ layer forms on the metal surface. It is worth noting that the intensities of peaks related to the iron phase diffraction (e.g., 44.9° and 65.93°) decrease in the absence of LBG, likely due to the blocking effect of the corrosion product layer. On the other hand, with LBG present, the same peaks exhibit a slight decrease in intensity, resulting from a thinner corrosion layer that enables X-rays to pass through and produce the diffraction peaks [6].

### 3.2. High Temperature (T = 80 °C)

The findings from the weight-loss experiment suggest that LBG can offer good protection even at higher temperatures, with the IE increasing as its concentration rises. The maximum IE was observed to be 44.59% after 24 h of immersion at 0.3 g L^−1^ LBG (Figure 1a). However, IE decreases at 80 °C, compared to those observed at room temperature. It has been reported that inhibitor molecules are continually adsorbed and desorbed on the metal surface, with this balance depending on temperature. For LBG, it appears that increasing the temperature favors the desorption of inhibitor molecules over their adsorption, resulting in a less stable adsorbed layer. These results are in line with those reported in the literature for polysaccharide corrosion inhibitors [2,3,4,16,18,27] and with the findings from the adsorption study reported in Section 2.2, confirming that LBG is mainly adsorbed via physical interactions with the metal surface. Locust bean gum (LBG) is a polysaccharide composed of mannose and galactose (e.g., M:G ratio is 4:1) that has low solubility in cold water. Its solubility depends on how its chains are arranged. In cold water, LBG adopts a random coil conformation. To uncoil its chains and improve its solubility, LBG must be heated at high temperature (e.g., up to 80 °C) [34,35]. Therefore, the high IE% observed at 80 °C may be due to the transition of its macromolecules from a coiled to an uncoiled state, increasing the number of adsorption groups available for adsorption on the metal surface. The magnitude of corrosion can be evaluated by estimating the thickness of the corrosion product layers after 24 h of exposure (Figure 10b,d). The images show that, without LBG, the corrosion product layer was thicker, ranging from 16 to 23 µm, than with LBG, which ranged from 5 to 7 µm. The GIXRD analysis presented in Figure 12b corroborates the SEM results. The peaks at 45.05° and 65.93°, associated with the ferritic phase, are clearly visible when the metal is in contact with the inhibited solution. However, these peaks nearly disappeared in the uninhibited solution, probably due to the blocking action of the corrosion product layer, which attenuates the X-ray beam [6]. Additionally, the top-view SEM analysis reveals differences between the two samples (Figure 10a,c). In the blank solution sample, FeCO_3_ crystals (EDS red square 2 in Figure S3b) developed on a Fe_3_C layer (EDS red square 1 in Figure S3a), whereas in the inhibited solution, an amorphous layer mainly composed of oxygen, carbon, and other elements from the steel composition (EDS in Figure S3c), no FeCO_3_ crystals are present (Figure 13c). The results align with the XRD patterns shown in Figure 12b. FeCO_3_ layers develop on steel when the concentrations of [Fe^2+^] and [${\mathrm{CO}}_{3}^{2-}$] surpass the solubility product limit [40,52,53,56]. High temperatures (e.g., above 60 °C) promote its formation (Figure 13d) [40,42,59]. The absence of FeCO_3_ indicates that LBG drastically reduces steel corrosion. Similar findings have also been documented in the literature. The authors noted that the inhibitor slowed the corrosion process, thus delaying the growth of the FeCO_3_ film [2,7,9,60,61].

The electrochemical experiments corroborate the gravimetric and morphological tests, showing a consistent increase in IE with increasing LBG concentration. Notably, at 80 °C, LBG exerted a greater influence on cathodic reactions than on the anodic reaction, indicating that LBG was more prone to adsorb on cathode sites. This adsorption likely hindered the HER, as explained further in Section 3.3. The Nyquist spectra show a similar pattern, with or without LBG: a large semicircle across all studied frequencies, with a consistent increase in *R*_ct_ as the LBG concentration increases (Figure 4c,d). The maximum IE was 55.81% after 24 h of immersion at 0.3 g L^−1^ LBG.

**Figure 14:**
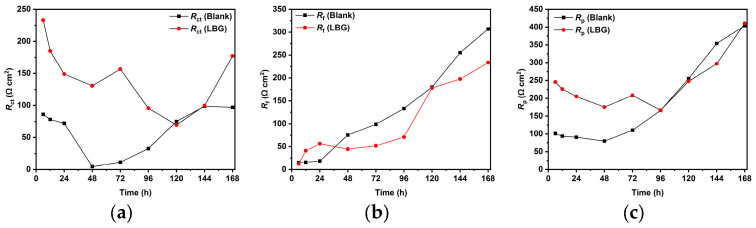
Variation in (**a**) *R*_ct_, (**b**) *R*_f_, and (**c**) *R*_p_, vs. time at 80 °C, without and with 0.3 g L^−1^ of LBG.

For both uninhibited and inhibited samples, weight loss experiments showed that the AWL decreased after 168 h, compared to the one observed after 24 h (Table S2), and that the LBG did not have any influence after a prolonged exposure at 80 °C. To understand the effect of the corrosion product layer on the adsorption of LBG on the metal surface, a series of EIS measurements at different immersion times was carried out, both with and without the optimal LBG concentration (e.g., 0.3 g L^−1^) (Figure 7 and Table S5). The data shows that the corrosion process can be divided into two stages, depending on the corrosion product developed: active-porous layer and mixed layer [56]. In the first stage, the bare sample undergoes rapid corrosion, forming a quick layer of corrosion products on its surface. The inductive loop observed at 25 °C for the free inhibitor disappears at 80 °C. As mentioned earlier, the inductive occurs as a result of the adsorption of FeOH_(ads)_ on the metal surface following metal corrosion. The quickly formed layer blocks adsorption sites for FeOH_(ads)_, leading to the disappearance of the inductive loop. This type of layer initially is a non-protective, amorphous layer (Figure 13c). Figure 14 shows the profile of *R*_ct_, *R*_f_, and *R*_p_ vs. time, with and without LBG. It can be seen from the images that, as the exposure time increases and the corrosive process continues (e.g., *R*_ct_ decreases over time), the precipitation of FeCO_3_ on the metal surface causes *R*_f_ (e.g., film resistance) to increase (Figure 13d). The polarization resistance (*R*_p_) (e.g., *R*_p_ = *R*_ct_ + *R*_f_) for the inhibited sample is consistently higher than that of the uninhibited sample for up to 72 h, with an IE of 47.04%. After 96 h (e.g., mixed layer stage, Figure 13e), the effect of LBG disappeared, and both samples showed a similar AWL. Up to this stage, both samples have formed a protective layer that can shield the metal from the aggressive solution. SEM analysis (Figure 11) and XRD analysis (Figure 12b) show that after 168 h, both samples were covered by a dense FeCO_3_ crystal layer. This stage is known as the mixed layer because, as shown in the cross-section analysis (Figure 11b,d), it involves the development of a double-layered structure. The inner layer is composed of Fe_3_C lamellar, formed at the beginning of the corrosion process, and amorphous FeCO_3_, precipitated in the lamellar structure (e.g., active-porous layer, Figure 13b,c). As the corrosion process continues, and [Fe^2+^] increases, depending on temperature, pH, and other factors, FeCO_3_ crystals form on the inner layer (Figure 13e) [53,62,63]. Interestingly, the mixed layer stage seems to be reached at different immersion times, with or without LBG. For example, for the blank sample, *R*_ct_ decreases initially (e.g., up to 48 h) and then begins to increase steadily, while *R*_f_ rises almost linearly with time throughout the experiment. SEM analysis shows that after 24 h (Figure 11a), the sample is only partially covered by a FeCO_3_ layer. Therefore, it is still susceptible to corrosion. However, after 48 h, *R*_ct_ begins to increase, indicating that a complete outer protective layer of FeCO_3_ has formed over the entire metal surface. On the other hand, the inhibited sample shows a longer active-porous layer stage. *R*_ct_ decreases up to 120 h and then begins to increase, while *R*_f_ is almost constant for the first 96 h of exposure. Notably, *R*_ct_ in the presence of LBG is always higher than the uninhibited sample, until it reaches a similar value after 120 h. These results indicate that the LBG reduced metal corrosion until a protective FeCO_3_ layer formed, after which its effectiveness was nullified.

### 3.3. Mechanism of Inhibition

**Figure 15:**
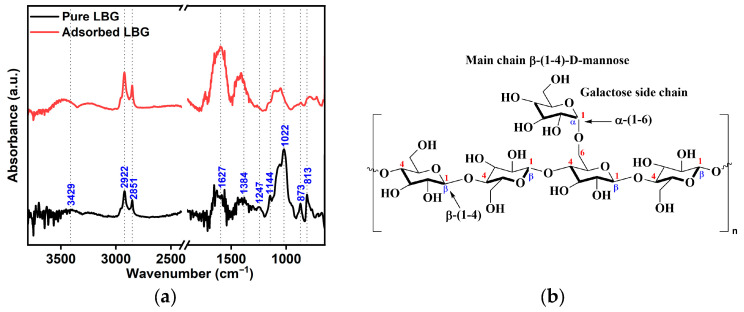
(**a**) FT-IR spectra of the native LBG and surface-adsorbed LBG. (**b**) chemical structure of LBG.

FTIR spectroscopy was used to understand key insights into how the functional groups of the LBG macromolecules interact with the metal surface. The combined FTIR spectra of native LBG and the LBG adsorbed on the metal surface after 24 h of immersion are shown in Figure 15a, and its structure is displayed in Figure 15b. The description of the peaks is provided in more detail in Table S6. The spectrum of the LBG adsorptive layer on the steel surface shows similar features to those observed in the native LBG spectrum. However, the peak intensities vary, and there is a shift within the same frequency range. This indicates a likely adsorption of LBG onto the metal surface [10]. The results align with previous research on the adsorption of polysaccharide inhibitors on steel surfaces. [3,4,8,10,16,36,64].

**Figure 16:**
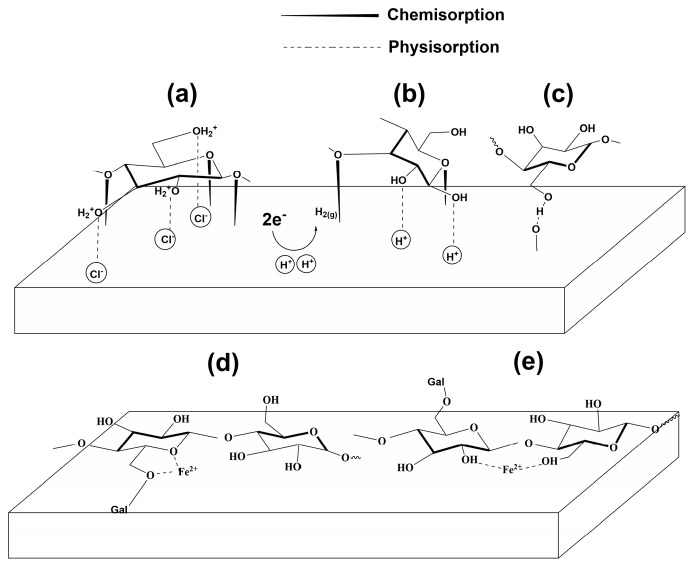
Schematic representation of the possible adsorption mechanism of LBG on N80 steel: (**a**) physisorption and chemisorption, (**b**,**c**) H-bond formation, and (**d**,**e**) chelating action.

The schematic model in Figure 16 illustrates possible adsorption mechanisms of LBG on the metal surface. This study demonstrates that LBG can reduce sweet corrosion at both tested temperatures, primarily through a combination of chemical and physical adsorption, with physical mechanisms playing a more significant role. Experimental results and literature review on the use of polysaccharide-like inhibitors reveal that their adsorption might mainly occur via: electrostatic interactions; unshared electron pair interactions; or, more likely, a combination of these mechanisms [2,3,4,27].

- Electrostatic adsorption

The results indicate that the adsorption process is mainly physical. Under acidic conditions, the many −OH groups in LBG molecules can be easily protonated. The inhibitor molecules are likely to interact with the metal surface through their protonated form, facilitated by chlorine ions adsorbed on the metal surface, as shown in Figure 16a [65].

- Chemical adsorption

From Figure 15a it is evident that the relative intensity of peaks related to glycosidic linkages such as β-(1-4) and α-(1-6) in the regions ranging between 1200–1000 cm^−1^ and 900–800 cm^−1^ decreased after the adsorption of LBG [66,67,68,69]. The lone pairs of electrons on endocyclic oxygen atoms in the glycosidic bonds, as well as in mannose and galactose units, can coordinate with the d-orbitals of Fe atoms to form bonds, as shown in Figure 16a,b.

- Adsorption through hydrogen bonds

Under the testing conditions (e.g., weak acid solution (pH = 3.5)), LBG molecules are only partially protonated, resulting in an equilibrium between protonated and neutral molecules. Therefore, LBG molecules can bind to the surface via hydrogen bonds between the unprotonated oxygen atom of their hydroxyl groups and the oxidized metal surface (Figure 16c) [3,4,45]. Moreover, studies on the use of guar gum (GG) as a corrosion inhibitor in acidic media suggested that the unprotonated hydroxyl groups may also interact with H^+^ ions formed at cathodic surface sites, helping stabilize the adsorption of the polysaccharide [17,22]. LBG has a structure similar to GG, with both having a mannose backbone chain, but they differ in the ratio of galactose [34,35]. The PDP measurements shown in Figure 3 indicate that LBG was able to inhibit the cathodic current density compared to the blank solution. Therefore, it may also be possible that LBG suppressed hydrogen formation at the cathodic sites via H-bond-assisted adsorption.

- Chelating Action

The changes observed in the FTIR (Figure 15a) plot between 1200 and 800 cm^−1^ (e.g., glycosidic linkages) may also be due to the formation of metal ion–polysaccharide complexes between the galactomannan macromolecules and the oxidized metal surface. The literature suggested two possible mechanisms of interaction: (i) through interactions between endocyclic and exocyclic lone pair electrons of O atoms of LBG molecules and the positively charged iron in siderite (Figure 16d) [17,22,70], and/or (ii) through Coulomb interactions involving partially negatively charged hydroxyl groups and the positively charged iron in siderite [71,72].

## 4. Experimental Procedure

### 4.1. Sample and Solution Preparation

This study was conducted on N80 carbon steel pipe with the composition reported in Table S7. The pipe was cut into coupons (e.g., 20 × 10 × 2 mm), ground and polished to achieve a mirror surface. Afterward, the specimens were cleaned first in distilled water and after in absolute alcohol, then dried.

The tested solution contained 2 wt.% KCl, prepared with analytical-grade KCl (Sigma-Aldrich, Poznań, Poland) and pure water (e.g., 0.055 µS cm^−1^). CO_2_ was bubbled for 2 h to purge O_2_. Once the specimens were installed, the electrochemical cell was sealed, and CO_2_ was bubbled into the solution for another 30 min. The CO_2_ bubbling continued during the test to reduce air contamination. The pH of the blank solution at 25 °C and 80 °C was 3.5 and 3.43, respectively. The tests were performed both with and without various LBG concentrations at 25 °C and 80 °C. Three coupons were used in the weight loss experiment, each with an exposed surface area of 5.2 cm^2^. The sample used in the electrochemical tests was embedded in PTFE, with one face exposed to the test solution (e.g., 0.50 cm^2^). Based on ASTM Standards G31 and G111, the minimum volume-to-exposure area ratio (V/A) for laboratory corrosion tests is 33 mL cm^−2^. Therefore, 1000 mL of the tested solution was added to the electrochemical cell to reach a V/A ratio that met or exceeded the recommended value. The V/A ratio was calculated as 62.11 mL cm^−2^.

### 4.2. Weight Loss Measurements

The weight-loss tests were performed by exposing the specimens to both the blank and the inhibited solution for 24 and 168 h, respectively. Prior to each, the samples were weighed with an analytical balance accurate to 0.1 mg. Once the immersion period ended, the samples were removed and washed with deionized water. Prior to re-weighing, the corrosion product was removed with a descaling solution [2], washed in distilled water and alcohol, then dried. The CR and IE% were determined using the following Equations (16) and (17) [4,73]:

(16) $$\mathrm{CR}\;(\mathrm{mm}y^{-1})=\frac{87.6∆m}{\mathrm{dAt}}$$

(17) $$\mathrm{IE}\;(\%)=\frac{\mathrm{CR}-CR^{\mathrm{inh}}}{\mathrm{CR}}\;\times \;100$$

where Δ*m* is the weight loss, *d* is the metal’s density, *A* represents the sample’s surface area, and *t* indicates immersion time. CR and CR^inh^ denote the corrosion rates of the steel without and with LBG, respectively.

All experiments were performed in triplicate, and the average results were calculated.

### 4.3. Electrochemical Measurements

The electrochemical tests were performed as previously described using a three-electrode cell (Gamry Instruments, Warminster, PA, USA). The setup included the sample (e.g., working electrode), and a counter electrode (e.g., platinum foil), and a reference electrode (e.g., saturated calomel electrode (SCE)). Measurements were performed with a Gamry Reference 600 potentiostat. Impedance spectroscopy (EIS) tests were carried out over a frequency range of 10 kHz to 10 mHz with an amplitude of 5 mV at open-circuit potential (OCP) after various immersion times. Potentiodynamic polarization (PDP) tests were carried out over a potential range of ±0.3 V vs. OCP and a scan rate of 1 mV s^−1^ after 24 and 168 h of immersion, with varying LBG concentrations. The EIS and PDP parameters were examined with Echem Analyst 5.21. The IE% was determined from the corrosion current density (*i*_corr_) and polarization resistances (*R*_p_) values, using Equations (18) and (19), respectively [6].

(18) $$\mathrm{IE}\;(\%)=\frac{i_{\mathrm{corr}}-i_{\mathrm{corr}}^{\mathrm{inh}}}{i_{\mathrm{corr}}}\times 100$$

(19) $$\mathrm{IE}\;(\%)=\frac{R_{p}^{\mathrm{inh}}-R_{p}}{R_{p}^{\mathrm{inh}}}\times 100$$

where $R_{p}^{\mathrm{inh}}$ and *R*_p_ are the polarization resistances and $i_{\mathrm{corr}}^{\mathrm{inh}}$ and *i*_corr_ corrosion current values in the presence and absence of LBG, respectively.

### 4.4. Surface Analysis

Morphological analysis (e.g., SEM-EDS (Tescan GmbH, Brno, Czech Republic) and GIXRD (Billerica, MA, USA) was performed on samples immersed for 24 and 168 h in the presence and absence of 0.3 g L^−1^ LBG at 25 °C and 80 °C. FTIR (Thermo Scientific Nicolet iS50, Indianapolis, IN, USA) analysis was conducted on samples immersed for 24 h in the presence or absence of 0.3 g L^−1^ LBG at 25 °C.

## 5. Conclusions

This study investigated the anti-corrosion effect of locust bean gum, a natural polysaccharide widely used as a thickening agent in the oil and gas sector, against 2 wt.% KCl solution saturated with CO_2_ at 25 °C and 80 °C. The results lead to the following conclusion:

- LBG demonstrated to be a strong and efficient corrosion inhibitor against CO_2_ corrosion, with its anticorrosive efficiency improving as concentration rose but declining with higher temperatures. EIS experiments showed that the highest inhibition efficiencies were 84.10% at 25 °C and 55.81% at 80 °C after 24 h of immersion, with 0.3 g L^−1^ of LBG.
- Long immersion experiments showed that the IE% at 25 °C stays steady throughout the experiment, with the IE reaching 83.97% after 168 h. However, at 80 °C and after 72 h (IE% = 47.04), LBG had no additional protective effect, due to the formation of a FeCO_3_ protective layer.
- PDP measurements indicate that LBG acts as a mixed-type inhibitor, reducing both cathodic and anodic reactions at both temperatures; however, at 80 °C, its suppression of the cathodic reaction was more pronounced.
- FTIR measurements show that LBG was strongly adsorbed onto the metal surface, with the adsorption process following the Temkin isotherm. The adsorption and activation data suggest that both physical and chemical mechanisms are involved in the process.
- SEM confirmed the efficacy of LBG as a corrosion inhibitor at both temperatures.

## Figures and Tables

**Table 1:** Adsorption parameters at 25 °C and 80 °C.

Temperature (°C)	*R* ^2^	*a*	*K*_ads_ (g L^−1^)	${∆G}_{\mathrm{ads}}^{{}^{\circ}}$ (kJ mol^−1^)
Langmuir				
25	0.995	-	24.39	−25.46
80	0.996	-	5.68	−23.23
Temkin				
25	0.973	−5.01	2721	−19.94
80	0.986	−3.49	81	−34.13

**Table 2:** *E*_a_ and *Q*_ads_ parameters calculated.

*C*_inh_ (g L^−1^)	*E*_a_ (kJ mol^−1^)	*Q*_ads_ (kJ mol^−1^)
Blank	12.16	-
0.05	20.00	−23.95
0.1	18.86	−15.67
0.2	18.10	−12.10
0.3	20.96	−15.48

**Table 3:** Potentiodynamic polarization parameters with and without concentrations of LBG after 24 h of immersion at 25 °C and 80 °C.

*C*_inh_ (g L^−1^)	−*β*_c_ (V dec^−1^)	*i*_corr_ (µA cm^−2^)	*E*_corr_ (V)	IE (%)
25 °C				
Blank	0.466 ± 0.042	37.09 ± 4.38	−0.657 ± 0.55	-
0.03	0.280 ± 0.038	16.52 ± 2.29	−0.646 ± 0.94	55.46
0.05	0.253 ± 0.025	7.54 ± 1.25	−0.630 ± 0.88	79.67
0.1	0.281 ± 0.035	6.05 ± 0.99	−0.633 ± 0.23	83.69
0.2	0.296 ± 0.033	5.13 ± 1.01	−0.637 ± 0.51	86.17
0.3	0.296 ± 0.033	4.69 ± 1.51	−0.650 ± 0.31	87.35
80 °C				
Blank	0.473 ± 0.041	171.15 ± 19.22	−0.707 ± 0.11	-
0.05	0.415 ± 0.053	155.70 ± 21.09	−0.700 ± 0.15	9.03
0.1	0.481 ± 0.044	126.43 ± 11.12	−0.705 ± 0.09	26.13
0.2	0.520 ± 0.059	110.01 ± 5.09	−0.699 ± 0.11	35.72
0.3	0.444 ± 0.031	90.17 ± 4.11	−0.711 ± 0.10	47.32

**Table 4:** EIS parameters in the absence and presence of different concentrations of LBG after 24 h of immersion time at 25 °C and 80 °C.

*C*_inh_ (g L^−1^)	*R*_s_ (Ω cm^2^)	*CPE*_f_		*R*_f_ (Ω cm^2^)	*CPE*_dl_		*R*_ct_ (Ω cm^2^)	*C*_dl_ (µF cm^−2^)	*L* (H cm^2^)	*R*_L_ (Ω cm^2^)	*R*_p_ (Ω cm^2^)	*χ*^2^ (×10^−4^)	IE (%)
		*Y*_f_ (s^n^ µΩ^−1^ cm^−2^)	*n*_f_		*Y*_dl_ (s^n^ µΩ^−1^ cm^−2^)	*n*_dl_							
						25 °C							
Blank	23.75 ± 3.41	-	-	-	510.51 ± 25.11	0.838 ± 0.06	574.72 ± 45.06	215.59	278.62 ± 85.06	150.33 ± 11.98	725.05	4.69	-
0.05	23.63 ± 2.10	80.39 ± 5.31	0.882 ± 0.05	57.65 ± 2.51	24.56 ± 2.40	0.982 ± 0.05	3119 ± 105.65	21.42	-	-	3176.65	3.77	77.18
0.1	28.26 ± 3.35	47.88 ± 3.95	0.867 ± 0.05	46.25 ± 2.05	32.15 ± 3.12	0.897 ± 0.05	3805 ± 88.56	14.37	-	-	3877.44	7.56	81.30
0.2	30.91 ± 3.21	46.44 ± 3.20	0.903 ± 0.09	72.44 ± 5.15	36.42 ± 3.41	0.889 ± 0.09	4514 ± 101.99	15.59	-	-	4560.25	3.63	84.10
0.3	21.73 ± 2.19	58.75 ± 4.44	0.862 ± 0.11	215.5 ± 13.43	13.81 ± 1.13	0.952 ± 0.06	4350 ± 115.15	9.17	-	-	4565.50	5.59	84.11
						80 °C							
Blank	11.38 ± 1.15	919.20 ± 25.66	0.907 ± 0.02	18.49 ± 0.41	621.40 ± 36.12	0.870 ± 0.06	72.13 ± 5.06	289.87	-	-	90.62	4.35	-
0.05	11.43 ± 1.01	769.20 ± 44.51	0.926 ± 0.05	22.43 ± 2.91	323.10 ± 35.23	0.860 ± 0.02	92.05 ± 3.65	127.32	-	-	114.48	1.19	20.83
0.1	10.75 ± 1.19	731.81 ± 46.11	0.907 ± 0.04	19.04 ± 2.11	219.81 ± 27.11	0.861 ± 0.02	106.11 ± 5.32	81.17	-	-	125.15	5.32	27.58
0.2	11.06 ± 1.26	686.84 ± 31.24	0.875 ± 0.05	15.01 ± 1.99	69.16 ± 5.88	0.88 ± 0.03	146.05 ± 9.11	25.74	-	-	161.51	0.66	45.25
0.3	11.98 ± 1.11	415.00 ± 28.06	0.931 ± 0.02	56.16 ± 9.01	189.10 ± 42.13	0.965 ± 0.06	148.90 ± 12.19	151.11	-	-	205.06	1.36	55.81

## Data Availability

The original contributions presented in the study are included in the article; further inquiries can be directed to the corresponding author.

## Supplementary Materials

The following supporting information can be downloaded at: https://www.mdpi.com/article/10.3390/molecules30234534/s1, Figure S1: EDS analysis of the tested steel surface after 24 h of immersion at 25 °C: (a) polished, (b) without, and (c) with 0.3 g L^−1^ of LBG solution. Figure S2: EDS analysis of the tested steel surface after 168 h of immersion at 25 °C: (a,b) red square 1, and red square 2, respectively, for blank solution; (c,d) red square 1, and red square 2, respectively, with 0.3 g L^−1^ of LBG solution. Figure S3: EDS analysis of the tested steel surface after 24 h of immersion at 80 °C: (a,b) red square 1, and red square 2, respectively, for blank solution; (c) with 0.3 g L^−1^ of LBG solution. Figure S4: EDS analysis of the tested steel surface after 168 h of immersion at 80 °C: (a) without, and (b) with 0.3 g L^−1^ of LBG solution. Figure S5: Microstructure of the N80 pipeline steel sample. Table S1: Average corrosion rate and inhibition efficiency obtained from weight loss measurements with and without different concentrations of LBG after 24 h of immersion at 25 °C and 80 °C. Table S2: Average corrosion rate and inhibition efficiency obtained from weight loss measurements at 0.3 g L^−1^ of LBG after 24 and 168 h of immersion at 25 °C and 80 °C. Table S3: Comparison of the reported inhibition efficiency of LBG and other natural corrosion inhibitors in different media with that of the present inhibitor, calculated via weight-loss measurements. Table S4: EIS parameters in the absence and presence of 0.3 g L^−1^ of LBG at 25 °C, and different immersion times. Table S5: EIS parameters in the absence and presence of 0.3 g L^−1^ of LBG at 80 °C, and different immersion times. Table S6: FTIR spectral analysis. Table S7: Chemical composition of the examined carbon steel (wt.%).
