# Supplementary material for: Locust Bean Gum: A Natural Polysaccharide as an Eco-Friendly Corrosion Inhibitor for N80 Carbon Steel in CO2-Saturated Saline Solution, Useful for the Oil and Gas Industry
Source: Molecules. 2025 Nov 24;30(23):4534. doi: 10.3390/molecules30234534 (PMC12693648; doi:10.3390/molecules30234534)
Supplement: Supplementary file 1 [file molecules-30-04534-s001.zip › molecules-3992388-supplementary.pdf]

molecules

Locust Bean Gum: A Natural Polysaccharide as an Eco-Friendly Corrosion Inhibitor for N80 Carbon Steel in CO_2-Saturated Saline Solution, Useful for the Oil and Gas Industry

Gaetano Palumbo[1,*], Marcin Górny[1], Dominika Święch[1], Adarsh Rai[2], Mahmoud M. Youssif[3,4]

[1]AGH University of Krakow, Faculty of Foundry Engineering, al. A. Mickiewicza 30, 30-059 Krakow, Poland
[2]AGH University of Krakow, Faculty of Metals Engineering and Industrial Computer Science, Mickiewicza 30, 30-059 Krakow, Poland
[3]AGH University of Krakow, Faculty of Non-Ferrous Metals, al. A. Mickiewicza 30, 30-059, Krakow, Poland
[4]Department of Chemistry, Faculty of Science, Tanta University, Tanta 31527, Egypt
Correspondence: gpalumbo@agh.edu.pl

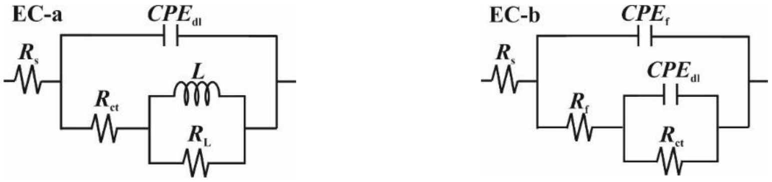

(a) (b)

Equivalent circuits used to fit experimental data. (a) without LBG at 25 °C; (b) with LBG at 25 °C, and without and with LBG at 80 °C.

Where R_s is the solution resistance, R_{ct} and CPE_{dl} are the charge transfer resistance and the constant phase element representing the double-charge layer capacitance, respectively. R_L and L are the inductive resistance and inductance, respectively. R_f and CPE_f are the resistance provided by the adsorbed inhibitor layer and its constant phase element, respectively.

The impedance of a *CPE* is described by Eq. S1, whereby Y_o is the double layer *CPE* coefficient, *j* is an imaginary number $(j = (-1)^{\frac{1}{2}}$, ω is the angular frequency in rad/s, and n (with value $-1 \leq n \leq 1$) is the phase shift, which also indicates the homogeneity (smoothness) of the corroding surface [1]. The double layer capacitance (C_{dl}) can be calculated as a function of R_s, R_{ct}, Y_o, and n, based on the model proposed by Brug et al. [2], according to Eq S2.

$$Z_{CPE} = Y_o[(j\omega)^n]^{-1} \tag{S1}$$

$$C_{dl} = Y_o^{\frac{1}{n}}\left[\frac{1}{R_s} + \frac{1}{R_{ct}}\right]^{\frac{n-1}{n}} \tag{S2}$$

$$C_{dl} = \frac{\varepsilon_0 \varepsilon A}{\delta} \tag{S3}$$

Where ε is the dielectric constant of the medium, ε_o is the vacuum permittivity, A is the electrode area, and δ is the thickness of the protective layer [3].

molecules

MDPI

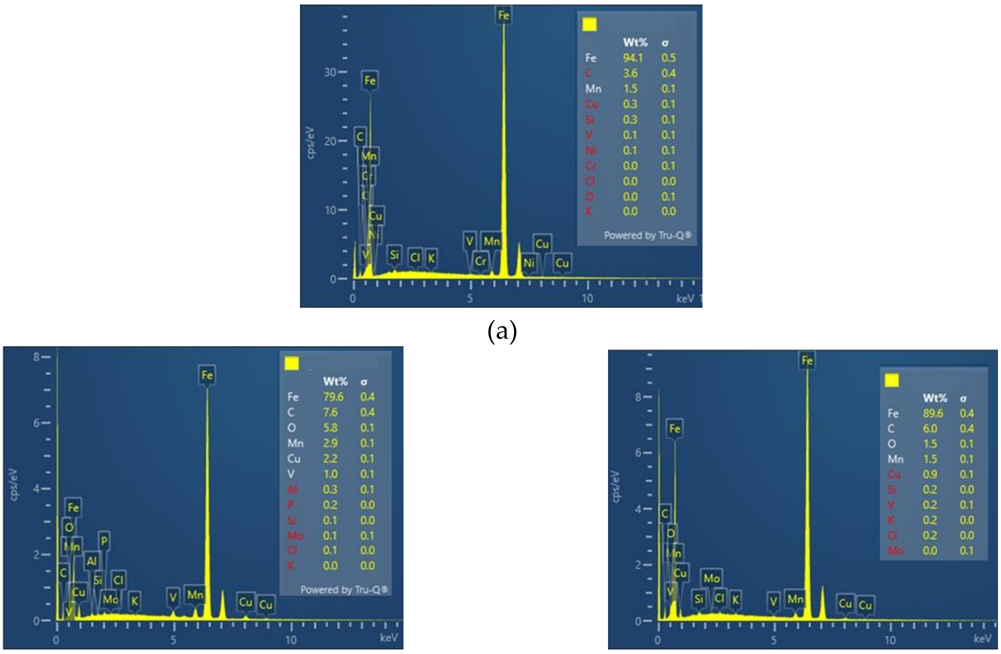

(a)

(b) (c)

Figure S1. EDS analysis of the tested steel surface after 24 h of immersion at 25 °C: (a) polished, (b) without, and (c) with 0.3 g L^{-1} of LBG solution.

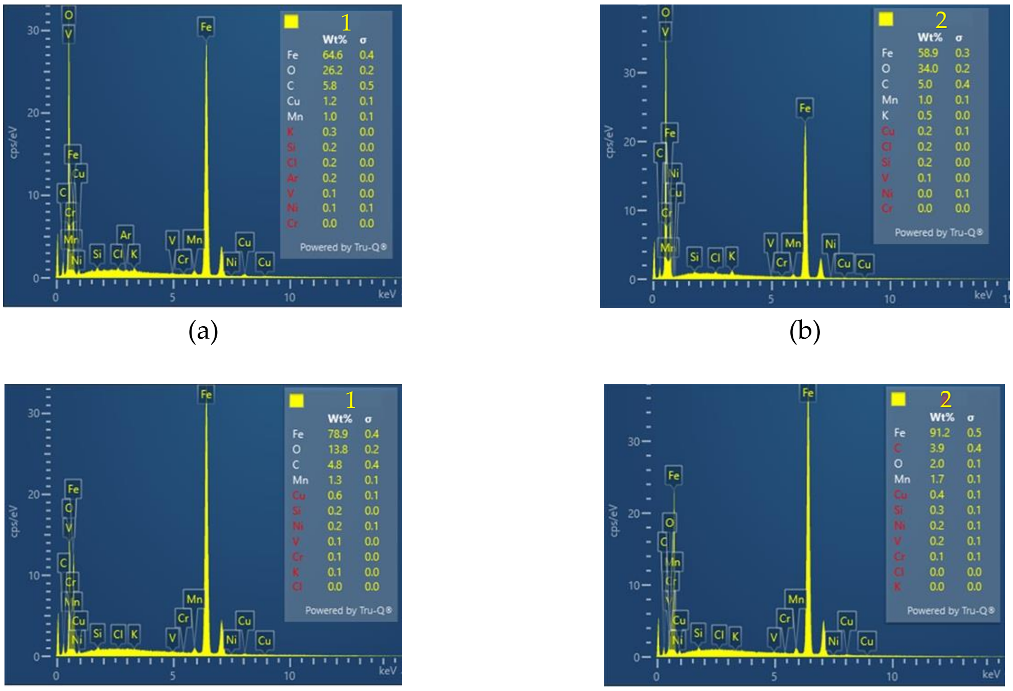

(a) (b)

(c)

Figure S2. EDS analysis of the tested steel surface after 168 h of immersion at 25 °C: (a-b) red square 1, and red square 2, respectively, for blank solution; (c-d) red square 1, and red square 2, respectively, with 0.3 g L^{-1} of LBG solution.

molecules

MDPI

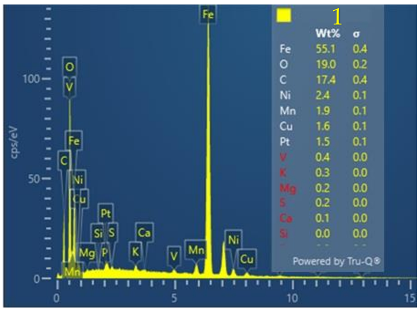

(a)

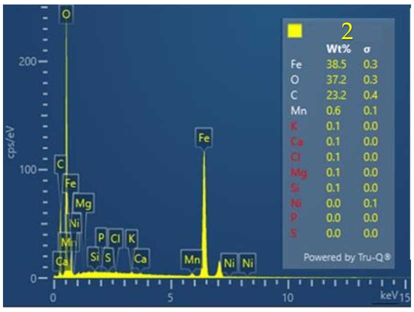

(b)

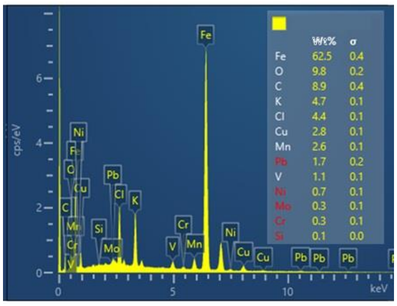

(c)

Figure S3. EDS analysis of the tested steel surface after 24 h of immersion at 80 °C: (a-b) red square 1, and red square 2, respectively, for blank solution; (c) with 0.3 g L^{-1} of LBG solution.

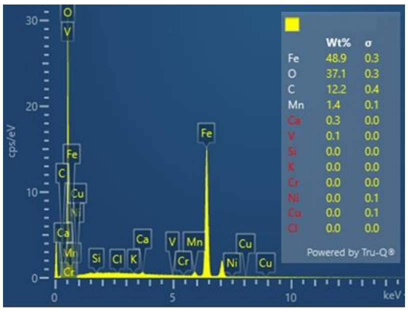

(a)

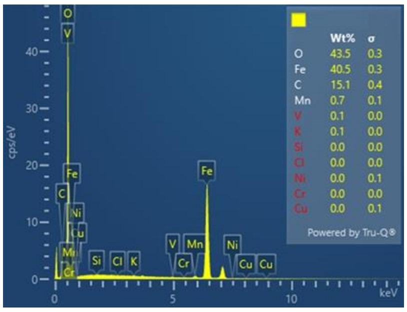

(b)

Figure S4. EDS analysis of the tested steel surface after 168 h of immersion at 80 °C: (a) without, and (b) with 0.3 g L^{-1} of LBG solution.

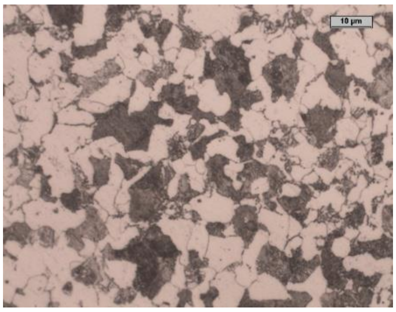

Figure S5. Microstructure of the N80 pipeline steel sample.

Table S1. Average corrosion rate and inhibition efficiency obtained from weight loss measurements with and without different cocetrations of LBG after 24 hours of immersion at 25 °C and 80 °C.

C_{inh} (g L^{-1})	CR (mg cm^{-2} h^{-1})	IE (%)	CR (mg cm^{-2} h^{-1})	IE (%)
	25 °C		80 °C	
Blank	0.082±0.009	-	0.176±0.011	-
0.05	0.041±0.008	50	0.144±0.009	18.18
0.1	0.037±0.008	54.88	0.121±0.010	31.25
0.2	0.034±0.009	61.77	0.106±0.012	39.77
0.3	0.026±0.005	68.29	0.097±0.008	44.59

Table S2. Average corrosion rate and inhibition efficiency obtained from weight loss measurements at 0.3 g L^{-1} of LBG after 24 and 168 hours of immersion at 25 °C and 80 °C.

C_{inh} (g L^{-1})	CR (mg cm^{-2} h^{-1})	IE (%)	CR (mg cm^{-2} h^{-1})	IE (%)
	25 °C		80 °C	
		24h		
Blank	0.082±0.009	-	0.176±0.011	-
0.3	0.026±0.005	68.20	0.097±0.008	44.59
		168h		
Blank	0.151±0.012	-	0.107±0.011	-
0.3	0.041±0.002	72.85	0.100±0.006	6.54

molecules

MDPI

Table S3. Comparison of the reported inhibition efficiency of LBG and other natural corrosion inhibitors in different media with that of the present inhibitor, calculated via weight-loss measurements.

**Inhibitors**	**Metal**	**Corrosive media**	**Inhibitor concentration (g L^{-1})**	**Temperature (°C)**	**IE (%)**	**Time of exposure (h)**	**Reference**
Xanthan gum	Low Carbon Steel	1 M HCl	1	30/60	74.24/55.40	6	[4]
Xanthan gum	Carbon Steel	15% HCl	0.4	25/60	90.20/60.57	6	[5]
Locust bean gum	stainless steel AISI 304	0.15 M NaCl	2.0	25	88.55	0.5	[6]
Locust bean gum	Carbon Steel	1 M HCl	1.0	25	87.72	0.5	[7]
Carboxymethyl cellulose	API 5L X60 Steel	3.5% NaCl in CO_2	1.5	25	39	1	[8]
Chitosan	API 5L X60 Steel	3.5% NaCl in CO_2	1	25	45	1	[8]
Guar gum	Carbon Steel (N80)	0.5 M KCl in CO_2	0.4	25/50	74.55/63.13	24	[9]
Gum arabic	Carbon Steel (N80)	0.5 M KCl in CO_2	0.5	25/45	68.78/40.74	24	[3]
Xanthan gum	Carbon Steel (N80)	0.5 M KCl in CO_2	1.0	30/90	55.11/49.68	24	[10]
Xanthan gum	Carbon Steel (N80)	0.5 M KCl in CO_2	1.0	30/90	44.27/28.97	72	[10]
Locust bean gum	Carbon Steel (N80)	0.5 M KCl in CO_2	0.3	25/80	68.20/44.59	24	Present work
Locust bean gum	Carbon Steel (N80)	0.5 M KCl in CO_2	0.3	25/80	87.22*/47.04*	72	Present work
Locust bean gum	Carbon Steel (N80)	0.5 M KCl in CO_2	0.3	25/80	72.85/6.54	168	Present work

* EIS experiments.

Table S4. EIS parameters in the absence and presence of 0.3 g L^{-1} of LBG at 25 °C, and different immersion times.

Time (h)	R_s (Ω cm^2)	CPE_f		R_f (Ω cm^2)	CPE_{dl}		R_{ct} (Ω cm^2)	C_{dl} (µF cm^{-2})	L (H cm^2)	R_L (Ω cm^2)	R_p (Ω cm^2)	χ^2 (× 10^{-4})	IE (%)
		Y_f (s^n µΩ^{-1} cm^{-2})	n_f		Y_{dl} (s^n µΩ^{-1} cm^{-2})	n_{dl}							
**Blank 25 °C**													
6	21.19	100	0.909	16.01	143	0.895	600	72	-	-	616.52	30.82	-
12	27.28	-	-	-	369	0.835	667	147	149	154	821	28.52	-
24	23.75	-	-	-	510	0.838	574	215	278.62	150	724	46.73	-
48	24.88	-	-	-	814.5	0.835	471	373	224.00	158	629	48.40	-
72	24.58	-	-	-	1060	0.869	398	606	124.60	100	498	23.98	-
96	22.59	-	-	-	1657	0.814	344	770	107.7	160	504	29.34	-
168	19.57	-	-	-	2642	0.848	257	1533	109.20	167	424	16.42	-
**LBG 25 °C**													
6	22.03	50.70	0.875	244.91	9.03	0.963	3919	6.51	-	-	4163.91	5.16	85.19
12	21.45	53.47	0.873	228.22	12.52	0.946	4187	7.83	-	-	4415.22	4.42	81.41
24	21.73	58.75	0.862	215.51	13.81	0.952	4350	9.17	-	-	4560.25	5.59	84.10
48	23.37	61.70	0.851	228.39	14.53	0.968	4398	11.16	-	-	4626.39	6.91	86.40
72	19.69	66.37	0.851	107.19	34.52	0.938	3791	21.31	-	-	3898.19	9.19	87.22
96	19.60	66.45	0.853	70.16	60.08	0.909	3302	30.57	-	-	3372.16	8.79	85.05
168	25.50	63.83	0.900	56.60	64.00	0.904	2588	32.34	-	-	2644,60	15.38	83.97

Table S5. EIS parameters in the absence and presence of 0.3 g L^{-1} of LBG at 80 °C, and different immersion times

Time (h)	R_s (Ω cm^2)	CPE_f		R_f (Ω cm^2)	CPE_{dl}		R_{ct} (Ω cm^2)	C_{dl} (µF cm^{-2})	L (H cm^2)	R_L (Ω cm^2)	R_p (Ω cm^2)	χ^2 (× 10^{-4})	IE (%)
		Y_f (s^n µΩ^{-1} cm^{-2})	n_f		Y_{dl} (s^n µΩ^{-1} cm^{-2})	n_{dl}							
**Blank 80 °C**													
6	10.26	342.0	0.971	14.98	225.7	0.936	86.03	148	-	-	101.01	7.32	-
12	9.86	509.4	0.996	15.66	371.4	0.967	78.11	305	-	-	93.77	10.29	-
24	11.38	919.6	0.907	18.49	621.4	0.870	72.13	290	-	-	90.62	5.62	-
48	11.23	1123	0.893	75.28	540x10^3	0.998	4.50	540x10^3	-	-	79.78	5.19	-
72	10.55	4483	0.871	98.97	337x10^3	0.998	11.34	33x10^3	-	-	110.31	3.86	-
96	10.50	5360	0.815	133.1	149x10^3	0.998	32.79	149x10^3	-	-	165.89	11.69	-
120	10.93	4091	0.811	180.2	124x10^3	0.998	74.96	125x10^3	-	-	255.16	17.87	-
144	12.5	2998	0.822	255.2	95x10^3	0.998	98.63	95x10^3	-	-	353.83	12.38	-
168	11.15	2785	0.829	306.8	66x10^3	0.998	96.91	66x10^3	-	-	430.71	16.43	-
**LBG 80 °C**													
6	13.22	287.90	0.869	12.6	170.3	0.980	233.00	150.38	-	-	245.60	14.08	58.87
12	12.61	361.60	0.930	41.05	199.00	0.944	185.00	138.96	-	-	226.05	14.11	58.52
24	11.98	415.70	0.931	56.16	189.10	0.965	148.90	151.11	-	-	205.6	1.36	55.81
48	12.80	509.50	0.976	44.57	300.00	0.988	130.60	280	-	-	175.17	8.89	54.45
72	14.90	897.40	0.927	51.71	414.01	0.929	156.60	417	-	-	208.31	7.86	47.04
96	12.11	1281	0.942	70.76	611.21	0.929	95.50	486	-	-	165.76	4.43	-0.08
120	14.82	2136	0.869	178.1	486x10^3	0.998	69.54	487x10^3	-	-	247.64	7.67	-3.04
144	17.31	2149	0.877	197.5	180x10^3	0.998	99.51	180x10^3	-	-	297.01	16.36	-19
168	20.04	2110	0.827	233.40	154x10^3	0.980	177.20	157x10^3	-	-	410.60	21.18	-4.89

Table S6. FTIR spectral analysis

Peak (cm^{-1})	Assigned To	Ref.
3429	Stretching vibrations bound to galactose and mannose of –OH	[11,12]
2922 and 2851	Antisymmetric and symmetric C-H stretching vibrations of the methylene hydroxyl groups ($-CH_2OH$)	[11,13]
1627	Vibration of C-C from the bond between C atoms of manonose and galactose	[12]
1384	Vibration mode of –CH	[11,13]
1247	Deformational vibrations of CH_2 and -C-OH	[11,13]
1148 and 1022	Stretching vibration of $-CH_2-O-CH_2-$ on the glycosidic linkage	[11,13]
873 and 813	Glycosidic linkages, attributed to α-D-galactopyranose and β-D-mannopyranose units	[13-15]

Table S 7. Chemical composition of the examined carbon steel (wt.%).

Element	Wt.%
C	0.39
Si	0.26
Cu	0.26
Mn	1.80
V	0.19
Cr	0.04
Ni	0.04
Al	0.03
Fe	96.99

References

1. Obot, I.B.; Onyeachu, I.B.; Umoren, S.A. Alternative corrosion inhibitor formulation for carbon steel in CO_2-saturated brine solution under high turbulent flow condition for use in oil and gas transportation pipelines. *Corros. Sci.* **2019**, *159*, 108140, doi:https://doi.org/10.1016/j.corsci.2019.108140.
2. Brug, G.J.; van den Eeden, A.L.G.; Sluyters-Rehbach, M.; Sluyters, J.H. The analysis of electrode impedances complicated by the presence of a constant phase element. *J. Electroanal. Chem. & Interfacial. Electrochem.* **1984**, *176*, 275-295, doi:https://doi.org/10.1016/S0022-0728(84)80324-1.
3. Palumbo, G.; Górny, M.; Banaś, J. Corrosion Inhibition of Pipeline Carbon Steel (N80) in CO_2-Saturated Chloride (0.5 M of KCl) Solution Using Gum Arabic as a Possible Environmentally Friendly Corrosion Inhibitor for Shale Gas Industry. *J. Mater. Eng. Perform.* **2019**, *28*, 6458-6470, doi:10.1007/s11665-019-04379-3.
4. Mobin, M.; Rizvi, M. Inhibitory effect of xanthan gum and synergistic surfactant additives for mild steel corrosion in 1M HCl. *Carbohydr. Polym.* **2016**, *136*, 384-393, doi:https://doi.org/10.1016/j.carbpol.2015.09.027.
5. Biswas, A.; Pal, S.; Udayabhanu, G. Experimental and theoretical studies of xanthan gum and its graft co-polymer as corrosion inhibitor for mild steel in 15% HCl. *Appl. Surf. Sci.* **2015**, *353*, 173-183, doi:http://dx.doi.org/10.1016/j.apsusc.2015.06.128.
6. Büyüksağiş, A.; Baydır, A.T.; Dilek, M. Locust Bean Gum as Corrosion Inhibitors in NaCl Solution. *Prot. Met. Phys. Chem. Surf.* **2021**, *57*, 211-221, doi:10.1134/S2070205120060076.
7. Abbout, S.; Zouarhi, M.; Chebabe, D.; Damej, M.; Berisha, A.; Hajjaji, N. Galactomannan as a new bio-sourced corrosion inhibitor for iron in acidic media. *Heliyon* **2020**, *6*, e03574, doi:10.1016/j.heliyon.2020.e03574.
8. Umoren, S.A.; AlAhmary, A.A.; Gasem, Z.M.; Solomon, M.M. Evaluation of chitosan and carboxymethyl cellulose as ecofriendly corrosion inhibitors for steel. *Int. J. Biol. Macromol.* **2018**, *117*, 1017-1028, doi:https://doi.org/10.1016/j.ijbiomac.2018.06.014.

9. Palumbo, G.; Święch, D.; Górny, M. Guar Gum as an Eco-Friendly Corrosion Inhibitor for N80 Carbon Steel under Sweet Environment in Saline Solution: Electrochemical, Surface, and Spectroscopic Studies. *Int. J. Mol. Sci.* **2023**, *24*, 12269.
10. Palumbo, G. Xanthan Gum as an Eco-Friendly Corrosion Inhibitor for N80 Carbon Steel Under High Pressure and High Temperature in Saline CO_2-Saturated Solution. *Materials* **2025**, *18*, 4450, doi:https://doi.org/10.3390/ma18194450
11. Giridharaprasad, S.; Jadhav, H.B.; Shewale, S.; Annapure, U. Study on chemical modification of locust bean gum for enhanced functionality. *J. Indian Chem. Soc.* **2024**, *101*, 101360, doi:https://doi.org/10.1016/j.jics.2024.101360.
12. Hadinugroho, W.; Martodihardjo, S.; Fudholi, A.; Riyanto, S. Esterification of citric acid with locust bean gum. *Heliyon* **2019**, *5*, doi:10.1016/j.heliyon.2019.e02337.
13. Thombare, N.; Mahto, A.; Singh, D.; Chowdhury, A.R.; Ansari, M.F. Comparative FTIR Characterization of Various Natural Gums: A Criterion for Their Identification. *Journal of Polymers and the Environment* **2023**, *31*, 3372-3380, doi:10.1007/s10924-023-02821-1.
14. Busch, V.M.; Pepa, L.S.; Panizzolo, L.A.; Buera, M.d.P.; Ferreira, F. Effect of Ultrasound and Enzymatic Hydrolysis on the Physicochemical Properties of Neltuma Ruscifolia Seed Gum and Other Galactomannan Gums. *Food and Bioprocess Technology* **2024**, *17*, 2380-2392, doi:10.1007/s11947-023-03258-z.
15. Yuen, S.-N.; Choi, S.-M.; Phillips, D.L.; Ma, C.-Y. Raman and FTIR spectroscopic study of carboxymethylated non-starch polysaccharides. *Food Chem.* **2009**, *114*, 1091-1098, doi:https://doi.org/10.1016/j.foodchem.2008.10.053.
